# Crystal structure of 9,10-bis­(1,3-di­thiol-2-yl­idene)-9,10-di­hydro­anthracene

**DOI:** 10.1107/S2056989015020800

**Published:** 2015-11-07

**Authors:** Yi Ren, Semin Lee, Jeffery A. Bertke, Jeffrey S. Moore

**Affiliations:** a Department of Chemistry, University of Illinois, Urbana, Illinois 61801, USA

**Keywords:** crystal structure, tetra­thia­fulvalene, TTF, exTTF, Wittig–Horner reaction

## Abstract

The crystal structure of the well-studied 9,10-bis­(1,3-di­thiol-2-yl­idene)-9,10-di­hydro­anthracene mol­ecule, C_20_H_12_S_4_, (I), also known as exTTF, is reported. The mol­ecular structure of (I) consists of a di­hydro­anthracene moiety with two 1,3-di­thiol-2-yl­idene substituents. This is a saddle-shaped mol­ecule, which inter­acts with a close neighbor through various π–π and C—H⋯π contacts to form a ‘dimer’. These ‘dimers’ inter­act through a series of C—H⋯S and C—H⋯π contacts to construct a complex three-dimensional extended structure.

## Abstract 2

The title complex is composed of saddle-shaped mol­ecules which closely inter­act in a pairwise fashion through π–π and C—H⋯π contacts to form ‘dimers’. These ‘dimers’ further inter­act through C—H⋯S and C—H⋯π contacts to construct a complex three-dimensional extended structure.

## Chemical context   

Since the first report on 9,10-bis­(1,3-di­thiol-2-yl­idene)-9,10-di­hydro­anthracene (exTTF) (I) as a highly-conjugated electron donor (Bryce & Moore, 1988 ▸), numerous studies have been conducted on the development of exTTF derivatives that are applicable toward organic electronics. (Brunetti *et al.*, 2012 ▸) To our surprise, the single crystal structure of exTTF has not been reported and most of the existing literature on exTTF focuses on theoretical calculations and modeling. (Gruhn *et al.*, 2006 ▸; Zhao & Truhlar, 2008 ▸) Herein, we report the single-crystal structure of exTTF.
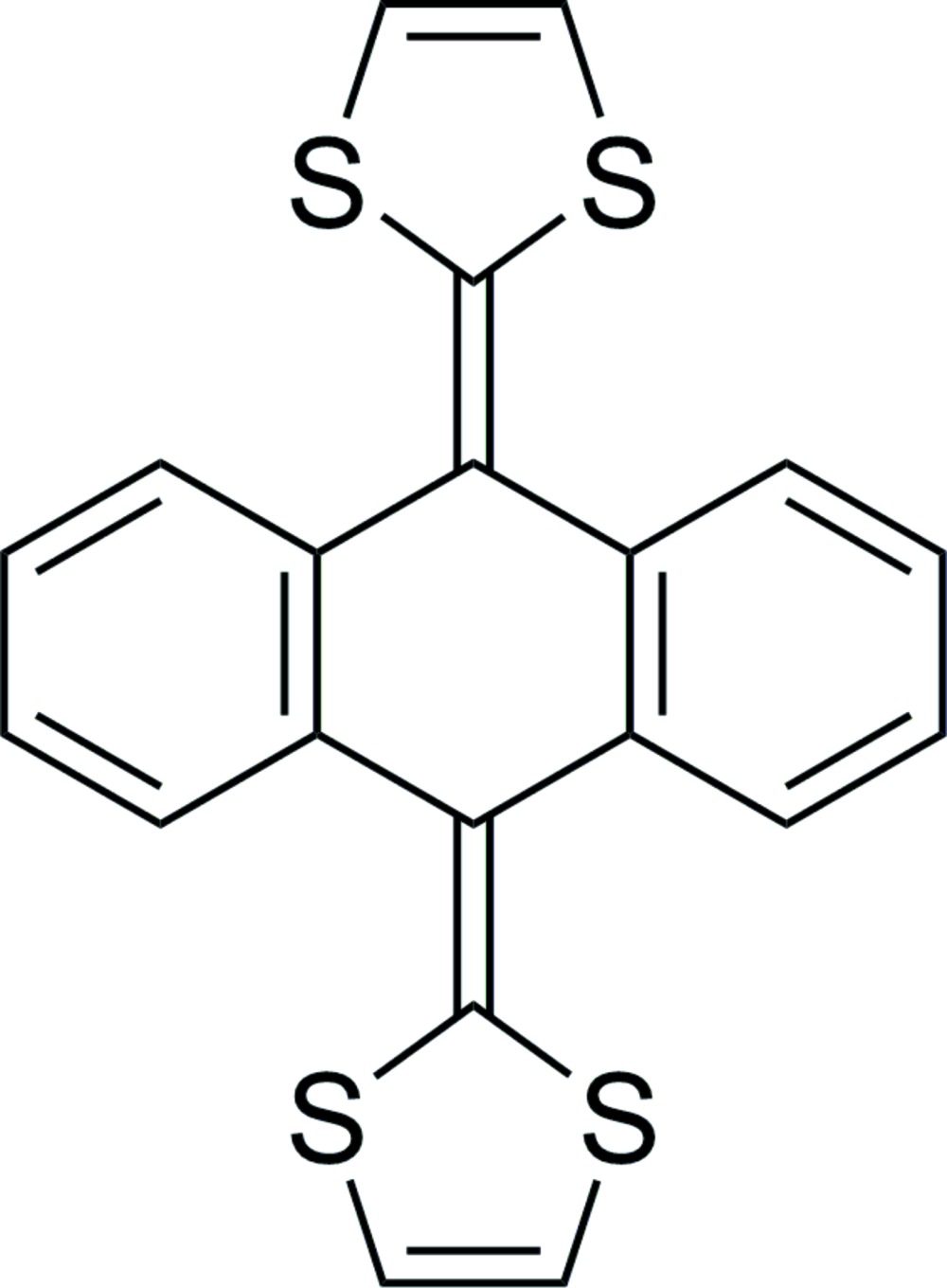


## Structural commentary   

**Figure 1:**
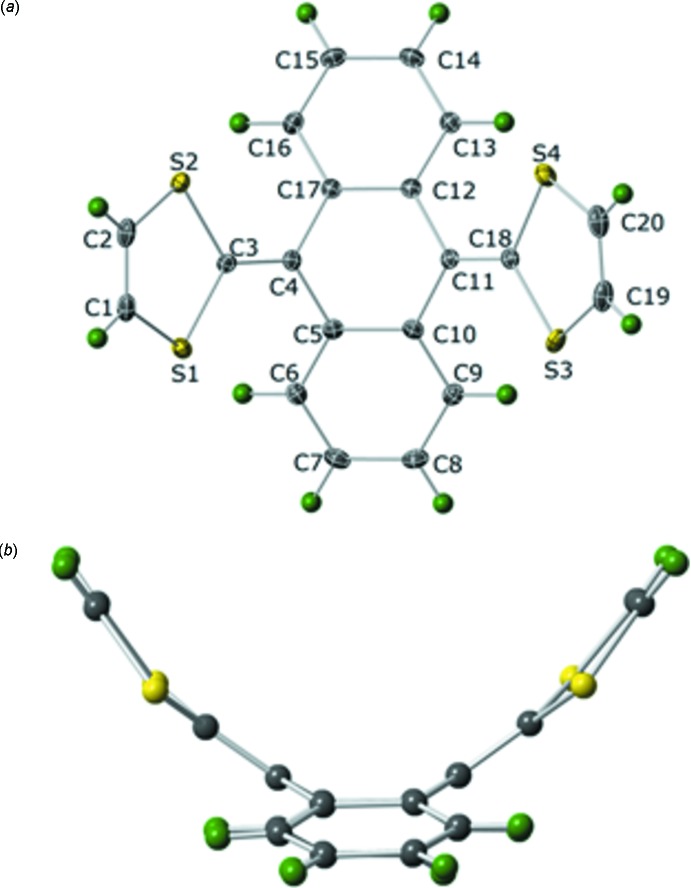
(*a*) Plot showing 35% probability ellipsoids for non-H atoms and circles of arbitrary size for H atoms for (I). (*b*) A view of (I) showing the saddle shape of the mol­ecule.

The mol­ecular structure of (I) consists of a di­hydro­anthracene moiety with 1,3-di­thiol-2-yl­idene groups substituted at the 9 and 10 positions, Fig. 1 ▸ *a*. The mol­ecule is saddle shaped in that the 1,3-di­thiol-2-yl­idene groups bend significantly up out of the plane of the central ring and the two benzene rings of di­hydro­anthracene moiety bend down out of the plane, Fig. 1 ▸ *b*. The central six-membered ring (C4–C5–C10–C11–C12–C17) is in a boat conformation in which the 1,3-di­thiol-2-yl­idene-substituted carbon atoms [C4 and C11] are bent out of the plane defined by C5, C10, C12, and C17. The torsion angles C10—C12—C17—C4 = 17.97 (12)° and C17—C5—C10—C11 = 17.22 (16)° for these two carbon atoms are quite similar.

The benzene rings bend out of the C5–C10–C12–C17 plane; the dihedral angle between this plane and the plane of the C5–C6–C7–C8–C9–C10 ring is 17.72 (15)° while the dihedral angle for the C12–C13–C14–C15–C16–C17 ring is 20.14 (13)°. The 1,3-di­thiol-2-yl­idene groups are bent more sharply out of the C5-C10-C12-C17 plane as evidenced by the torsion angles C3—C4—C5—C10 τ = 138.06 (15)° and C18—C11—C12—C17 τ = 139.23 (15)°. The five-membered rings both adopt an envelope conformation with the carbon atom bonded to the di­hydro­anthracene [C3 and C18] being the one puckered out of the plane. The torsion angles C3—S1—C1—C2 τ = −8.09 (14)° and C18—S4—C20—C19 τ = −6.65 (15)° show that the bend in each ring is fairly similar.

The average C—C bond length within the benzene rings (excluding the edges shared with the central ring) is 1.391 Å as is typical of phenyl rings. The length of the edges shared with the central ring are slightly longer C5—C10 = 1.419 (2) Å and C12—C17 = 1.412 (2) Å. The remaining C—C distances making up the central ring are longer still with an average of 1.477 Å. Since the distances within the central ring are in between those of typical C—C single and double bonds; this supports the idea of a highly delocalized bonding motif throughout the di­hydro­anthracene ring system. The bond distances between the di­hydro­anthracene and the 1,3-di­thiol-2-yl­idene groups are on the order of typical C=C bonds, C3=C4 = 1.360 (2) Å and C11=C18 = 1.361 (2) Å.

## Supra­molecular features   

**Figure 2:**
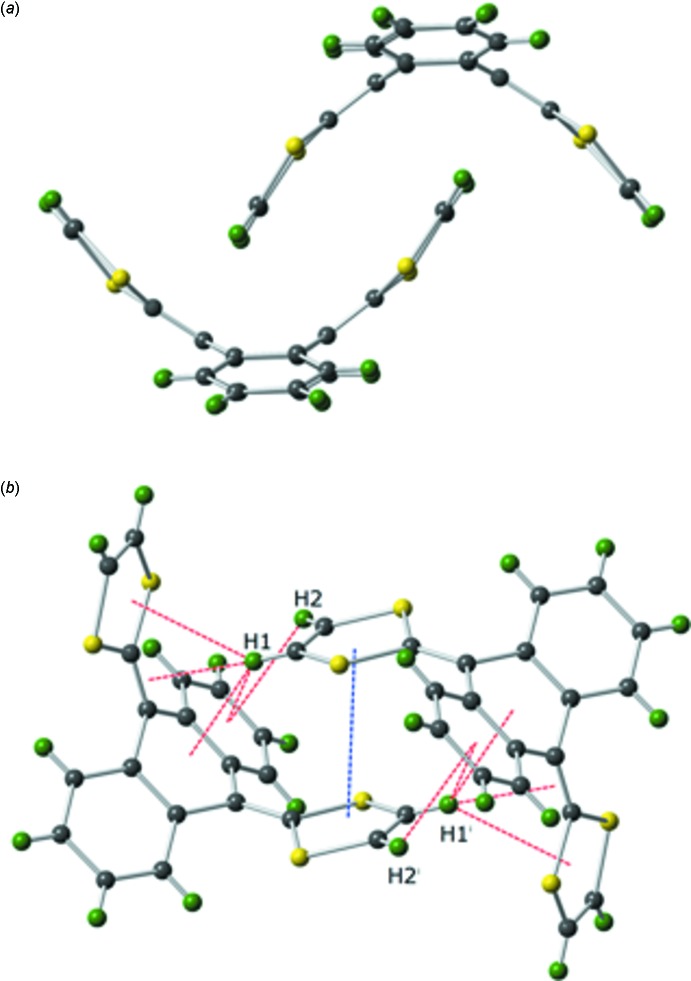
A view of a ‘dimer’ of (I) showing (*a*) how the 1,3-di­thiol-2-yl­idene group of one mol­ecule sits in the U-shape of a neighboring mol­ecule, and (*b*) the inter­actions between the mol­ecules that make up the ‘dimer’. Gray = Carbon, yellow = Sulfur, green = Hydrogen, blue dashed line = π–π inter­action, red dashed line = C—H⋯π inter­action. [Symmetry operation: (i) −*x* + 1, −*y* + 1, −*z* + 1.]

Through a series of C—H⋯π and π–π inter­actions, each mol­ecule of (I) closely inter­acts with a neighboring mol­ecule to form a ‘dimer’, Fig. 2 ▸. The π–π inter­action is between the C1–C2–S2–C3–S1 ring and the C1^i^–C2^i^–S2^i^–C3^i^–S1^i^ ring [symmetry operation: (i) −*x* + 1, −*y* + 1, −*z* + 1] and is rather long at 4.068 (15) Å. There are five C—H⋯π inter­actions between the two mol­ecules in which atoms H1 and H2 of one mol­ecule inter­act with various π systems of the neighbor. The shortest contact is between H1 and the C11^i^=C18^i^ double bond at 2.606 (12) Å [H1⋯C11^i^ 2.686 (19) Å; H1⋯C18^i^ 2.700 (14) Å]. There is another short contact between H1 and the central ring of the di­hydro­anthracene, H1⋯centroid (C4^i^–C5^i^–C10^i^–C11^i^–C12^i^–C17^i^) 2.852 (11) Å. Two other C—H⋯π inter­actions involve H1; H1⋯centroid (C18^i^–S3^i^–C19^i^–C20^i^–S4^i^) 3.167 (11) Å, and H1⋯centroid (C5^i^–C6^i^–C7^i^–C8^i^–C9^i^–C10^i^) 3.553 (15) Å. The fifth inter­action between the ‘dimer’ mol­ecules is H2⋯centroid (C5^i^–C6^i^–C7^i^–C8^i^–C9^i^–C10^i^) 3.222 (12) Å.

**Figure 3:**
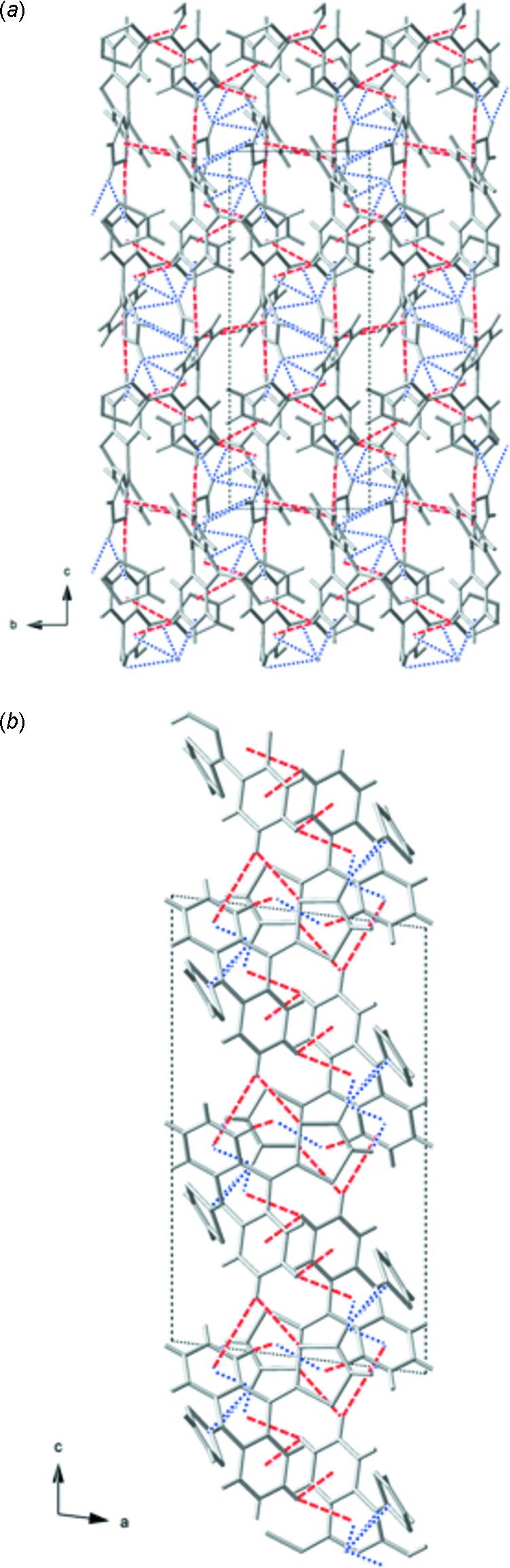
A portion of the two-dimensional network formed between ‘dimers’ when only the di­hydro­anthracene CH inter­actions are taken into account viewed (*a*) along the *a* axis, and (*b*) along the *b* axis. Blue dashed lines are intra-dimer inter­actions and red dashed lines are di­hydro­anthracene CH inter-dimer inter­actions.

The ‘dimers’ of (I) inter­act through C—H⋯S and C—H⋯π contacts with neighboring mol­ecules to form a complex three-dimensional network. There are five C—H⋯S and C—H⋯π inter­actions in which the CH group involved resides on the di­hydro­anthracene portion of (I). H14 has inter­actions with two groups of a neighboring mol­ecule; one C—H⋯S contact H14⋯S1^ii^ 2.922 (12) Å and one C—H⋯π contact H14⋯centroid (C5^ii^–C6^ii^–C7^ii^–C8^ii^–C9^ii^–C10^ii^) 3.779 (17) Å [symmetry operation: (ii) *x*, 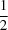 − *y*, −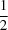 + *z*]. H15 also inter­acts with two groups on a neighboring mol­ecule through two C—H⋯π contacts; H15⋯centroid (C12^iii^–C13^iii^–C14^iii^–C15^iii^–C16^iii^–C17^iii^) 3.385 (17) Å and H15⋯centroid (C4^iii^–C5^iii^–C10^iii^–C11^iii^–C12^iii^–C17^iii^) 3.543 (14) Å [symmetry operation: (iii) −*x* + 1, −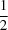 + *y*, 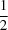 − *z*]. It should be noted that the mol­ecules generated by symmetry operations (ii) and (iii) form a ‘dimer’. The final inter­action involving a CH group on the di­hydro­anthracene is H6⋯centroid (S1^iv^–C1^iv^–C2^iv^–S2^iv^–C3^iv^) 2.865 (11) Å [symmetry operation: (iv) −*x* + 1, −*y*, −*z* + 1]. Taking these inter­actions into account, a two-dimensional layered structure is formed (Fig. 3 ▸) in which the layers extend along the *bc* plane.

**Figure 4:**
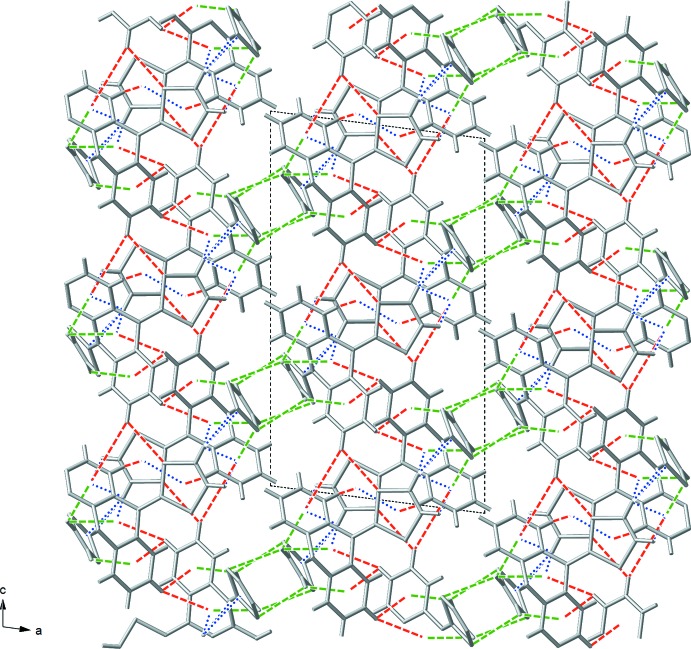
A portion of the three-dimensional structure of (I), viewed along the *b* axis, showing the two-dimensional layers connecting along the *a* axis. Blue dashed lines are intra-dimer inter­actions, red dashed lines are di­hydro­anthracene CH inter-dimer inter­actions, and green dashed lines are 1,3-di­thiol-2-yl­idene CH inter-dimer inter­actions.

There are also five C—H⋯S and C—H⋯π inter­actions in which the CH group involved resides on the 1,3-di­thiol-2-yl­idene portion of (I). H19 and H20 each inter­act with one neighboring mol­ecule through three C—H⋯π contacts; H19⋯centroid (C5^v^–C6^v^–C7^v^–C8^v^–C9^v^–C10^v^) 2.829 (18) Å, H19⋯centroid (C4^v^–C5^v^–C10^v^–C11^v^–C12^v^–C17^v^) 3.301 (11) Å, and H20⋯centroid (C12^v^–C13^v^–C14^v^–C15^v^–C16^v^–C17^v^) 2.767 (11) Å [symmetry operation: (v) *x*, *y* + 1, *z*]. These hydrogen atoms also inter­act with another mol­ecule *via* C—H⋯S contacts; H19⋯S4^vi^ 3.367 (12) Å and H20⋯S3^vi^ 3.288 (14) Å [symmetry operation: (vi) −*x*, *y* + 0.5, −*z* + 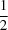]. When these inter­actions are taken into account, the two-dimensional layers are connected along the *a* axis to form a three-dimensional extended structure, Fig. 4 ▸.

## Database survey   

Many derivatives of (I) have been crystallographically characterized with various substituents on the di­hydro­anthracene, the di­thiol, or both moieties. A search of the Cambridge Crystal Database (CCD) (Groom & Allen, 2014 ▸) yields three derivatives of (I) with substituents on the di­hydro­anthracene and twelve derivatives with substituents on both the di­hydro­anthracene and the di­thiol. There have been twenty-nine structures reported in the CCD with substituents on the di­thiol ring. The complex most closely related to (I) is the tetra­methyl-substituted 9,10-anthracenediyl­idene-2,2′-bis­(4,5-dimethyl-1,3-di­thiole) (Bryce *et al.*, 1990 ▸; CCD code: JIJGIS). This mol­ecule crystallizes in the same space group as (I) (monoclinic, *P*2_1_/*c*) and has a similar saddle shape. It also appears to form similar ‘dimers’ in which there are both C—H⋯π and π–π inter­actions between the two mol­ecules.

A recent computational study focused on predicting the most energetically favored ‘dimers’ of (I) (Denis & Iribarne, 2015 ▸). This study predicted the ‘dimer’ characterized in (I) as the second most favorable, being 1.7 kcal mol^−1^ less stable than the predicted favorite. The study details π–π stacking between two of the di­thiol rings, C—H⋯π contacts between the di­thiol H atoms and the anthracene rings, π–π stacking between anthracene units, as well as an inter­action between the partial positive charge of the S atoms and the anthracene rings for the preferred computational ‘dimer’. The study briefly describes the C—H⋯π and π–π inter­actions found in (I), but states that the lack of π–π stacking between the anthracene moieties is the reason this orientation is slightly less favorable.

## Synthesis and crystallization   

**Figure 5:**
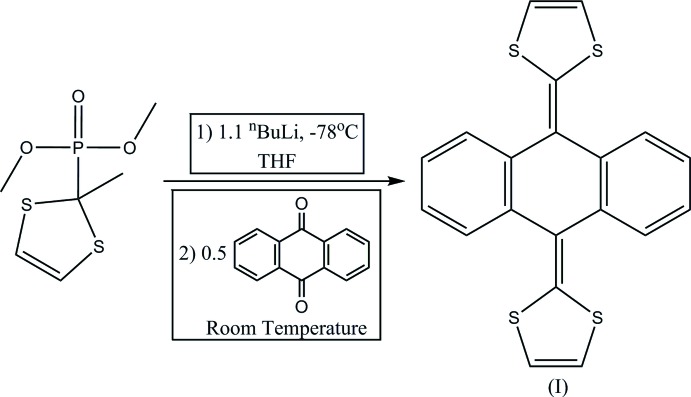
The synthesis of (I).

The title complex, 9,10-bis­(1,3-di­thiol-2-yl­idene)-9,10-di­hydro­anthracene (I), was synthesized following a literature procedure (Yamashita *et al.*, 1989 ▸), Fig. 5 ▸. X-ray quality crystals were grown from slow diffusion of chloro­form into a diethyl ether solution of (I).

## Refinement   

Crystal data, data collection and structure refinement details are summarized in Table 1 ▸. A structural model consisting of the target mol­ecule was developed. H atoms were included as riding idealized contributors with C–H = 0.95 Å *U*_iso_(H) = 1.2*U*_eq_(C).

## Supplementary Material

Crystal structure: contains datablock(s) I. DOI: 10.1107/S2056989015020800/bg2573sup1.cif

Structure factors: contains datablock(s) I. DOI: 10.1107/S2056989015020800/bg2573Isup2.hkl

Supporting information file. DOI: 10.1107/S2056989015020800/bg2573Isup3.cdx

Supporting information file. DOI: 10.1107/S2056989015020800/bg2573Isup4.cml

CCDC reference: 1434765

Additional supporting information:  crystallographic information; 3D view; checkCIF report

## Figures and Tables

**Table 1:** Experimental details

Crystal data	
Chemical formula	C_20_H_12_S_4_
*M*_r_	380.54
Crystal system, space group	Monoclinic, *P*2_1_/*c*
Temperature (K)	100
*a*, *b*, *c* (Å)	11.2759 (3), 7.6073 (2), 19.5596 (5)
β (°)	97.313 (1)
*V* (Å^3^)	1664.16 (8)
*Z*	4
Radiation type	Cu *K*α
μ (mm^−1^)	5.21
Crystal size (mm)	0.36 × 0.10 × 0.04

Data collection	
Diffractometer	Bruker APEXII CCD
Absorption correction	Integration (*SADABS*; Bruker, 2014 ▸)
*T*_min_, *T*_max_	0.999, 1.000
No. of measured, independent and observed [*I* > 2σ(*I*)] reflections	25822, 3056, 2757
*R*_int_	0.037

Refinement	
*R*[*F* ^2^ > 2σ(*F* ^2^)], *wR*(*F* ^2^), *S*	0.025, 0.061, 1.08
No. of reflections	3056
No. of parameters	218
H-atom treatment	H-atom parameters constrained
Δρ_max_, Δρ_min_ (e Å^−3^)	0.25, −0.25

Computer programs: *APEX2*, *SAINT* (Bruker, 2014 ▸), *XPREP* and *XCIF* (Bruker, 2014 ▸), *SHELXS2014* and *SHELXTL* (Sheldrick, 2008 ▸), *SHELXL2014* (Sheldrick, 2015 ▸), *CrystalMaker* (CrystalMaker, 1994 ▸), and *publCIF* (Westrip, 2010 ▸).

## supplementary crystallographic information

### Crystal data

**Table:**

C_20_H_12_S_4_	*F*(000) = 784
*M**_r_* = 380.54	*D*_x_ = 1.519 Mg m^−^^3^
Monoclinic, *P*2_1_/*c*	Cu *K*α radiation, λ = 1.54178 Å
*a* = 11.2759 (3) Å	Cell parameters from 9948 reflections
*b* = 7.6073 (2) Å	θ = 4.0–68.3°
*c* = 19.5596 (5) Å	µ = 5.21 mm^−^^1^
β = 97.313 (1)°	*T* = 100 K
*V* = 1664.16 (8) Å^3^	Needle, yellow
*Z* = 4	0.36 × 0.10 × 0.04 mm

### Data collection

**Table:**

Bruker APEXII CCD diffractometer	3056 independent reflections
Radiation source: microfocus sealed tube	2757 reflections with *I* > 2σ(*I*)
Multilayer mirrors monochromator	*R*_int_ = 0.037
profile data from φ and ω scans	θ_max_ = 68.4°, θ_min_ = 4.0°
Absorption correction: integration (*SADABS*; Bruker, 2014)	*h* = −13→13
*T*_min_ = 0.999, *T*_max_ = 1.000	*k* = −9→9
25822 measured reflections	*l* = −23→23

### Refinement

**Table:**

Refinement on *F*^2^	Hydrogen site location: inferred from neighbouring sites
Least-squares matrix: full	H-atom parameters constrained
*R*[*F*^2^ > 2σ(*F*^2^)] = 0.025	*w* = 1/[σ^2^(*F*_o_^2^) + (0.0242*P*)^2^ + 1.0937*P*] where *P* = (*F*_o_^2^ + 2*F*_c_^2^)/3
*wR*(*F*^2^) = 0.061	(Δ/σ)_max_ = 0.001
*S* = 1.08	Δρ_max_ = 0.25 e Å^−^^3^
3056 reflections	Δρ_min_ = −0.25 e Å^−^^3^
218 parameters	Extinction correction: *SHELXL2014* (Sheldrick, 2015), Fc^*^=kFc[1+0.001xFc^2^λ^3^/sin(2θ)]^-1/4^
0 restraints	Extinction coefficient: 0.00303 (14)

### Special details

**Table:**

Experimental. One distinct cell was identified using *APEX2* (Bruker, 2014). Fourteen frame series were integrated and filtered for statistical outliers using *SAINT* (Bruker, 2014) then corrected for absorption by integration using *SAINT*/*SADABS* v2014/2 (Bruker, 2014) to sort, merge, and scale the combined data. No decay correction was applied.
Geometry. All e.s.d.'s (except the e.s.d. in the dihedral angle between two l.s. planes) are estimated using the full covariance matrix. The cell e.s.d.'s are taken into account individually in the estimation of e.s.d.'s in distances, angles and torsion angles; correlations between e.s.d.'s in cell parameters are only used when they are defined by crystal symmetry. An approximate (isotropic) treatment of cell e.s.d.'s is used for estimating e.s.d.'s involving l.s. planes.
Refinement. Structure was phased by direct methods (Sheldrick, 2015). Systematic conditions suggested the unambiguous space group. The space group choice was confirmed by successful convergence of the full-matrix least-squares refinement on *F*^2^. The final map had no significant features. A final analysis of variance between observed and calculated structure factors showed little dependence on amplitude and resolution.

### Fractional atomic coordinates and isotropic or equivalent isotropic displacement parameters (Å^2^)

**Table:**

	*x*	*y*	*z*	*U*_iso_*/*U*_eq_	
S1	0.50450 (3)	0.25084 (5)	0.52454 (2)	0.01521 (11)	
S2	0.63047 (3)	0.26765 (5)	0.40288 (2)	0.01627 (11)	
S3	0.06123 (3)	0.70767 (5)	0.35517 (2)	0.01672 (11)	
S4	0.19863 (4)	0.71272 (5)	0.23764 (2)	0.01788 (11)	
C1	0.64924 (14)	0.3346 (2)	0.53580 (9)	0.0183 (3)	
H1	0.6861	0.3720	0.5798	0.022*	
C2	0.70600 (14)	0.3442 (2)	0.48073 (9)	0.0195 (4)	
H2	0.7846	0.3908	0.4834	0.023*	
C3	0.49073 (14)	0.24302 (19)	0.43367 (8)	0.0124 (3)	
C4	0.38454 (13)	0.23077 (18)	0.39233 (8)	0.0117 (3)	
C5	0.26881 (13)	0.22783 (19)	0.41988 (8)	0.0118 (3)	
C6	0.24960 (14)	0.1319 (2)	0.47833 (8)	0.0150 (3)	
H6	0.3121	0.0610	0.5009	0.018*	
C7	0.14057 (14)	0.1385 (2)	0.50395 (8)	0.0183 (3)	
H7	0.1295	0.0746	0.5444	0.022*	
C8	0.04781 (15)	0.2381 (2)	0.47066 (8)	0.0189 (3)	
H8	−0.0266	0.2435	0.4885	0.023*	
C9	0.06369 (14)	0.3300 (2)	0.41104 (8)	0.0152 (3)	
H9	−0.0010	0.3955	0.3877	0.018*	
C10	0.17326 (13)	0.32754 (19)	0.38499 (7)	0.0119 (3)	
C11	0.19641 (13)	0.42281 (19)	0.32222 (7)	0.0112 (3)	
C12	0.27915 (13)	0.33216 (19)	0.28095 (7)	0.0114 (3)	
C13	0.26370 (13)	0.3330 (2)	0.20902 (8)	0.0135 (3)	
H13	0.1987	0.3962	0.1849	0.016*	
C14	0.34196 (14)	0.2427 (2)	0.17217 (8)	0.0163 (3)	
H14	0.3304	0.2443	0.1232	0.020*	
C15	0.43704 (14)	0.1501 (2)	0.20707 (8)	0.0176 (3)	
H15	0.4917	0.0904	0.1820	0.021*	
C16	0.45236 (14)	0.1447 (2)	0.27850 (8)	0.0154 (3)	
H16	0.5165	0.0786	0.3020	0.018*	
C17	0.37462 (13)	0.23512 (19)	0.31631 (8)	0.0118 (3)	
C18	0.15426 (13)	0.5873 (2)	0.30614 (7)	0.0127 (3)	
C19	0.08465 (15)	0.9113 (2)	0.31802 (9)	0.0217 (4)	
H19	0.0541	1.0166	0.3352	0.026*	
C20	0.14664 (15)	0.9132 (2)	0.26496 (9)	0.0226 (4)	
H20	0.1622	1.0200	0.2426	0.027*	

### Atomic displacement parameters (Å^2^)

**Table:**

	*U*^11^	*U*^22^	*U*^33^	*U*^12^	*U*^13^	*U*^23^
S1	0.0144 (2)	0.0198 (2)	0.01084 (19)	0.00031 (14)	−0.00078 (15)	−0.00062 (14)
S2	0.01037 (19)	0.0225 (2)	0.0160 (2)	0.00129 (14)	0.00170 (15)	0.00215 (15)
S3	0.0145 (2)	0.0149 (2)	0.0206 (2)	0.00404 (14)	0.00183 (15)	−0.00264 (15)
S4	0.0217 (2)	0.0146 (2)	0.0173 (2)	0.00085 (15)	0.00232 (16)	0.00485 (15)
C1	0.0149 (8)	0.0181 (8)	0.0203 (8)	0.0026 (6)	−0.0042 (6)	−0.0033 (7)
C2	0.0129 (8)	0.0186 (8)	0.0252 (9)	0.0005 (6)	−0.0043 (7)	−0.0007 (7)
C3	0.0132 (7)	0.0113 (7)	0.0129 (7)	0.0022 (6)	0.0034 (6)	0.0008 (6)
C4	0.0132 (7)	0.0088 (7)	0.0133 (7)	0.0017 (6)	0.0015 (6)	0.0011 (6)
C5	0.0126 (7)	0.0101 (7)	0.0127 (7)	−0.0020 (6)	0.0015 (6)	−0.0031 (6)
C6	0.0163 (8)	0.0141 (7)	0.0142 (8)	−0.0006 (6)	0.0005 (6)	0.0004 (6)
C7	0.0206 (8)	0.0210 (8)	0.0137 (8)	−0.0052 (7)	0.0038 (6)	0.0032 (6)
C8	0.0160 (8)	0.0234 (8)	0.0188 (8)	−0.0024 (7)	0.0079 (6)	−0.0009 (7)
C9	0.0131 (7)	0.0158 (8)	0.0168 (8)	0.0000 (6)	0.0016 (6)	−0.0017 (6)
C10	0.0138 (7)	0.0112 (7)	0.0103 (7)	−0.0023 (6)	0.0004 (6)	−0.0023 (6)
C11	0.0086 (7)	0.0134 (7)	0.0112 (7)	−0.0010 (6)	−0.0005 (6)	−0.0013 (6)
C12	0.0112 (7)	0.0103 (7)	0.0128 (7)	−0.0030 (6)	0.0018 (6)	−0.0003 (6)
C13	0.0124 (7)	0.0143 (7)	0.0137 (8)	−0.0020 (6)	0.0004 (6)	0.0006 (6)
C14	0.0189 (8)	0.0193 (8)	0.0108 (7)	−0.0031 (6)	0.0024 (6)	−0.0016 (6)
C15	0.0187 (8)	0.0186 (8)	0.0166 (8)	0.0012 (6)	0.0066 (6)	−0.0053 (6)
C16	0.0137 (7)	0.0149 (8)	0.0176 (8)	0.0020 (6)	0.0019 (6)	−0.0011 (6)
C17	0.0117 (7)	0.0110 (7)	0.0125 (7)	−0.0021 (6)	0.0013 (6)	−0.0004 (6)
C18	0.0102 (7)	0.0159 (7)	0.0113 (7)	−0.0007 (6)	−0.0014 (6)	−0.0008 (6)
C19	0.0195 (8)	0.0126 (8)	0.0311 (10)	0.0023 (6)	−0.0046 (7)	−0.0020 (7)
C20	0.0239 (9)	0.0114 (7)	0.0299 (10)	−0.0005 (7)	−0.0064 (7)	0.0026 (7)

### Geometric parameters (Å, º)

**Table:**

S1—C1	1.7398 (16)	C8—C9	1.391 (2)
S1—C3	1.7654 (16)	C8—H8	0.9500
S2—C2	1.7471 (16)	C9—C10	1.395 (2)
S2—C3	1.7670 (16)	C9—H9	0.9500
S3—C19	1.7454 (17)	C10—C11	1.477 (2)
S3—C18	1.7647 (16)	C11—C18	1.361 (2)
S4—C20	1.7421 (17)	C11—C12	1.480 (2)
S4—C18	1.7680 (16)	C12—C13	1.396 (2)
C1—C2	1.323 (2)	C12—C17	1.412 (2)
C1—H1	0.9500	C13—C14	1.390 (2)
C2—H2	0.9500	C13—H13	0.9500
C3—C4	1.360 (2)	C14—C15	1.387 (2)
C4—C5	1.474 (2)	C14—H14	0.9500
C4—C17	1.477 (2)	C15—C16	1.386 (2)
C5—C6	1.396 (2)	C15—H15	0.9500
C5—C10	1.419 (2)	C16—C17	1.398 (2)
C6—C7	1.386 (2)	C16—H16	0.9500
C6—H6	0.9500	C19—C20	1.323 (3)
C7—C8	1.385 (2)	C19—H19	0.9500
C7—H7	0.9500	C20—H20	0.9500
			
C1—S1—C3	95.78 (8)	C9—C10—C11	123.73 (13)
C2—S2—C3	95.46 (8)	C5—C10—C11	117.26 (13)
C19—S3—C18	95.87 (8)	C18—C11—C10	123.34 (14)
C20—S4—C18	95.80 (8)	C18—C11—C12	121.89 (14)
C2—C1—S1	117.40 (12)	C10—C11—C12	114.52 (13)
C2—C1—H1	121.3	C13—C12—C17	119.07 (14)
S1—C1—H1	121.3	C13—C12—C11	122.78 (13)
C1—C2—S2	117.29 (13)	C17—C12—C11	118.10 (13)
C1—C2—H2	121.4	C14—C13—C12	120.97 (14)
S2—C2—H2	121.4	C14—C13—H13	119.5
C4—C3—S1	123.98 (12)	C12—C13—H13	119.5
C4—C3—S2	124.09 (12)	C15—C14—C13	119.83 (14)
S1—C3—S2	111.82 (8)	C15—C14—H14	120.1
C3—C4—C5	122.49 (14)	C13—C14—H14	120.1
C3—C4—C17	123.03 (14)	C16—C15—C14	120.03 (15)
C5—C4—C17	114.27 (13)	C16—C15—H15	120.0
C6—C5—C10	119.05 (14)	C14—C15—H15	120.0
C6—C5—C4	122.96 (13)	C15—C16—C17	120.83 (14)
C10—C5—C4	118.00 (13)	C15—C16—H16	119.6
C7—C6—C5	120.94 (14)	C17—C16—H16	119.6
C7—C6—H6	119.5	C16—C17—C12	119.25 (14)
C5—C6—H6	119.5	C16—C17—C4	123.32 (14)
C8—C7—C6	120.09 (15)	C12—C17—C4	117.40 (13)
C8—C7—H7	120.0	C11—C18—S3	124.44 (12)
C6—C7—H7	120.0	C11—C18—S4	123.16 (12)
C7—C8—C9	119.94 (15)	S3—C18—S4	112.16 (8)
C7—C8—H8	120.0	C20—C19—S3	117.33 (13)
C9—C8—H8	120.0	C20—C19—H19	121.3
C8—C9—C10	120.92 (14)	S3—C19—H19	121.3
C8—C9—H9	119.5	C19—C20—S4	117.59 (13)
C10—C9—H9	119.5	C19—C20—H20	121.2
C9—C10—C5	119.01 (14)	S4—C20—H20	121.2
			
C3—S1—C1—C2	−8.09 (14)	C18—C11—C12—C13	−43.5 (2)
S1—C1—C2—S2	−1.21 (19)	C10—C11—C12—C13	142.00 (14)
C3—S2—C2—C1	9.82 (14)	C18—C11—C12—C17	139.23 (15)
C1—S1—C3—C4	−162.04 (13)	C10—C11—C12—C17	−35.31 (18)
C1—S1—C3—S2	14.06 (9)	C17—C12—C13—C14	−1.2 (2)
C2—S2—C3—C4	161.58 (14)	C11—C12—C13—C14	−178.48 (14)
C2—S2—C3—S1	−14.51 (9)	C12—C13—C14—C15	0.0 (2)
S1—C3—C4—C5	1.8 (2)	C13—C14—C15—C16	1.4 (2)
S2—C3—C4—C5	−173.83 (11)	C14—C15—C16—C17	−1.6 (2)
S1—C3—C4—C17	176.22 (11)	C15—C16—C17—C12	0.3 (2)
S2—C3—C4—C17	0.6 (2)	C15—C16—C17—C4	178.43 (15)
C3—C4—C5—C6	−41.9 (2)	C13—C12—C17—C16	1.0 (2)
C17—C4—C5—C6	143.19 (14)	C11—C12—C17—C16	178.43 (13)
C3—C4—C5—C10	138.06 (15)	C13—C12—C17—C4	−177.19 (13)
C17—C4—C5—C10	−36.82 (18)	C11—C12—C17—C4	0.2 (2)
C10—C5—C6—C7	−2.6 (2)	C3—C4—C17—C16	42.7 (2)
C4—C5—C6—C7	177.45 (14)	C5—C4—C17—C16	−142.47 (14)
C5—C6—C7—C8	1.5 (2)	C3—C4—C17—C12	−139.19 (15)
C6—C7—C8—C9	0.7 (2)	C5—C4—C17—C12	35.66 (18)
C7—C8—C9—C10	−1.7 (2)	C10—C11—C18—S3	−2.8 (2)
C8—C9—C10—C5	0.6 (2)	C12—C11—C18—S3	−176.84 (11)
C8—C9—C10—C11	−179.69 (14)	C10—C11—C18—S4	171.04 (11)
C6—C5—C10—C9	1.5 (2)	C12—C11—C18—S4	−3.0 (2)
C4—C5—C10—C9	−178.52 (13)	C19—S3—C18—C11	163.72 (13)
C6—C5—C10—C11	−178.22 (13)	C19—S3—C18—S4	−10.70 (9)
C4—C5—C10—C11	1.8 (2)	C20—S4—C18—C11	−163.82 (13)
C9—C10—C11—C18	40.0 (2)	C20—S4—C18—S3	10.68 (9)
C5—C10—C11—C18	−140.37 (15)	C18—S3—C19—C20	6.74 (14)
C9—C10—C11—C12	−145.59 (14)	S3—C19—C20—S4	−0.06 (19)
C5—C10—C11—C12	34.08 (18)	C18—S4—C20—C19	−6.65 (15)
